# Analgesic Efficacy of Intraoperative Superior Hypogastric Plexus (SHP) Block during Abdominal Hysterectomy: A Systematic Review and Meta-Analysis of Controlled Trials

**DOI:** 10.3390/medicina59050893

**Published:** 2023-05-06

**Authors:** Hany Salem, Ibtihal Abdulaziz Bukhari, Maha Al Baalharith, Nasser AlTahtam, Safa Alabdrabalamir, Mohammed Ziad Jamjoom, Saeed Baradwan, Ehab Badghish, Mohammed Abuzaid, Fatimah Shakir AbuAlsaud, Osama Alomar, Abdullah Alyousef, Ahmed Abu-Zaid, Ismail Abdulrahman Al-Badawi

**Affiliations:** 1 Department of Obstetrics and Gynecology, King Faisal Specialist Hospital and Research Center, Riyadh, Saudi Arabia; 2 Clinical Sciences Department, College of Medicine, Princess Nourah bint Abdulrahman University, Riyadh, Saudi Arabia; 3 Department of Obstetrics and Gynecology, Urogynecology Division, Ministry of National Guard—Health Affairs, Riyadh, Saudi Arabia; 4 Department of Anesthesia, Obstetric Anesthesia Section, Ministry of National Guard—Health Affairs, Riyadh, Saudi Arabia; 5 Riyadh Second Health Cluster, Riyadh, Saudi Arabia; 6 Department of Obstetrics and Gynecology, King Fahad Armed Forces Hospital, Jeddah, Saudi Arabia; 7 Department of Obstetrics and Gynecology, King Faisal Specialist Hospital and Research Center, Jeddah, Saudi Arabia; 8 Department of Obstetrics and Gynecology, Maternity and Children Hospital, Makkah, Saudi Arabia; 9 Department of Obstetrics and Gynecology, Muhayil General Hospital, Muhayil, Saudi Arabia; 10 College of Medicine, Almaarefa University, Riyadh, Saudi Arabia; 11 College of Graduate Health Sciences, University of Tennessee Health Science Center, Memphis, TN 38163, USA

**Keywords:** superior hypogastric plexus, hysterectomy, postoperative pain, opioid, meta-analysis

## Abstract

*Background and Objectives:* Abdominal hysterectomy is a major surgery that is often associated with pronounced postsurgical pain. The objective of this research is to conduct a systematic review and meta-analysis of all randomized controlled trials (RCTs) and nonrandomized comparative trials (NCTs) that have surveyed the analgesic benefits and morbidity of intraoperative superior hypogastric plexus (SHP) block (intervention) compared with no SHP block (control) during abdominal hysterectomy. *Materials and Methods:* The Cochrane Central Register of Controlled Trials (CENTRAL), Google Scholar, Web of Science, PubMed, Scopus, and Embase were searched from inception until 8 May 2022. The Cochrane Collaboration tool and Newcastle–Ottawa Scale were used to evaluate the risk of bias of RCTs and NCTs, respectively. In a random effects mode, the data were pooled as risk ratio (RR) or mean difference (MD) with 95% confidence interval (CI). *Results:* Five studies (four RCTs and one NCT) comprising 210 patients (SHP block = 107 and control = 103) were analyzed. The overall postsurgical pain score (n = 5 studies, MD = −1.08, 95% CI [−1.41, −0.75], *p* < 0.001), postsurgical opioid consumption (n = 4 studies, MD = −18.90 morphine milligram equivalent, 95% CI, [−22.19, −15.61], *p* < 0.001), and mean time to mobilization (n = 2 studies, MD = −1.33 h, 95% CI [−1.98, −0.68], *p* < 0.001) were significantly decreased in the SHP block group contrasted with the control arm. Nevertheless, there was no significant variance between both arms regarding operation time, intraoperative blood loss, postsurgical NSAID consumption, and hospital stay. There were no major side effects or sympathetic block-related aftermaths in both groups. *Conclusions:* During abdominal hysterectomy and receiving perioperative multimodal analgesia, the administration of intraoperative SHP block is largely safe and exhibits better analgesic effects compared to cases without administration of SHP block.

## 1. Introduction

Abdominal hysterectomy is a major surgery that is often associated with pronounced postsurgical pain [1]. Insufficient control of postsurgical pain is bridged to a wide array of adverse aftermaths, one of which is dependence on large doses of nonsteroidal anti-inflammatory drugs (NSAIDs) and opioids [1,2].

The root of postsurgical pain after abdominal hysterectomy may arise from visceral and somatic pain origins [3]. The somatic pain instigates from pain receptors localized to epidermis as well as deep tissues of the abdominal wall. This somatic pain is often controlled with administration of abdominal wall blocks and/or wound infiltrations [1]. On the other hand, the origin of visceral pain is generally difficult to precisely pinpoint, owing to the intricacy of visceral nociceptive mechanisms [4]. Nevertheless, the current understanding is that pelvic visceral pain instigates from a chief autonomic innervation provided by the superior hypogastric plexus (SHP) [5]. Therefore, neurectomy or blockade of the SHP is endorsed as a credible scheme to lessen various causes of chronic pelvic pain [6]. Nevertheless, the role of SHP to control postsurgical pain after abdominal hysterectomy is not fully elucidated [1,3].

A restricted number of comparative studies has probed the benefits of intraoperative SHP block on enhancing postsurgical analgesia and minimizing opioid intake during abdominal hysterectomy [1,3,7,8,9]. Nevertheless, the findings of these studies have not yet been systematically and meta-analytically summarized. Such research is paramount to generate conclusions that can solidly guide clinical choices.

The goal of the present research is to summarize the evidence from randomized and nonrandomized comparative investigations that surveyed the analgesic benefits and morbidity of intraoperative SHP block during abdominal hysterectomy.

## 2. Materials and Methods

### 2.1. Study Protocol

The study protocol of this exempted research was not retrospectively registered and it was completed according guidelines of the Preferred Reporting Items for Systematic Reviews and Meta-Analyses (PRISMA) statement [10] and the Cochrane Handbook for Systematic Reviews of Interventions [11].

### 2.2. Eligibility Criteria

The eligibility criteria encompassed: (i) patients—individuals undergoing abdominal hysterectomy; (ii) intervention—SHP block; (iii) comparator—no SHP block; (iv) outcomes—postsurgical pain, postsurgical opioid consumption, postsurgical opioid, NSAID consumption, rescue analgesic time, operation time, amount of intraoperative bleeding, length of hospitalization, time to first bowel movement or urinary passage, time to first mobilization, and adverse events; and (v) study design—published randomized controlled trials (RCTs) and nonrandomized comparative trials (NCTs). The exclusion criteria encompassed: non-original studies, single-arm investigations, and trials using minimally invasive hysterectomy.

### 2.3. Databases, Search Strategy, and Study Selection Process

The Cochrane Central Register of Controlled Trials (CENTRAL), Google Scholar, Web of Science, PubMed, Scopus, and Embase were searched from inception until 8 May 2022. Supplementary Table S1 illustrates the search strategy employed in all databases. The studies were selected in a two-fold process comprising screening of titles/abstracts and followed by reading the full texts of the relevant studies. The reference lists of eligible studies were checked for potential additional studies. Two coauthors independently completed database search and study selection, and inconsistencies were sorted out by discussion.

### 2.4. Data Items, Study Quality Assessment, and Data Collection Process

The following baseline characteristics of the included studies were extracted: last author’s name, date of publication, country of publication, study groups, sample size of patients, age of patients, body mass index of patients, and details of the intervention (SHP block) and control (no SHP block) groups. The primary outcomes of this investigation comprised postsurgical pain (according to the 10-point visual analogue scale (VAS) scoring system) at different time points (0, 2, 6, 12, 24, and 48 h after surgery) and total postsurgical opioid intake (quantified using morphine milligram equivalent (MME) unit). The secondary outcomes of this investigation comprised postsurgical NSAID consumption (mg), NSAID rescue analgesic time (min), duration of surgery (min), amount of intraoperative bleeding (mL), time to first mobilization (h), time to first bowel movement or urinary passage (h), length of hospital stay (d), and adverse events (%).

The Cochrane risk of bias assessment tool for RCTs [12] and the Newcastle–Ottawa Scale for NCTs [13] were employed to evaluate study quality.

All relevant data were collected according to a predetermined form. Four pairs of coauthors independently completed the data collection, and inconsistencies were sorted out by discussion among the pairs.

### 2.5. Data Analysis

Using the random effects (DerSimonian and Laird) model [14], the continuous and dichotomous data were summarized as mean difference (MD) and risk ratio (RR), respectively, with 95% confidence interval (CI). Heterogeneity was confirmed based on the Cochran’s Q test *p* value < 0.1 [15] and Higgin’s I^2^ > 50% [16]. The Review Manager Software (version 5.4.0 for Windows) was employed to produce graphical forest plots. The stability of the summary results were tested via leave-one-out sensitivity analyses, whereas publication bias was tested via funnel plots for asymmetry and Egger’s regression test [17]. The STATA Software (version 17.0 for Windows) was employed to produce the graphical forest plots for the leave-one-out sensitivity analyses and funnel plots for publication bias. Statistical significance was determined as a two-tailed *p* value < 0.05. Some outcomes did not report the required mean and standard deviation values, and they were computed from other parameters (e.g., medians, ranges (minimum–maximum), or interquartile ranges) as described previously by Wan et al. [18]. Some outcomes were reported only qualitatively when meta-analysis was not feasible because of the small number of studies, or computation of effect size was not reliable based on the presented data.

## 3. Results

### 3.1. Summary of Literature Search and Included Studies

**Figure 1:**
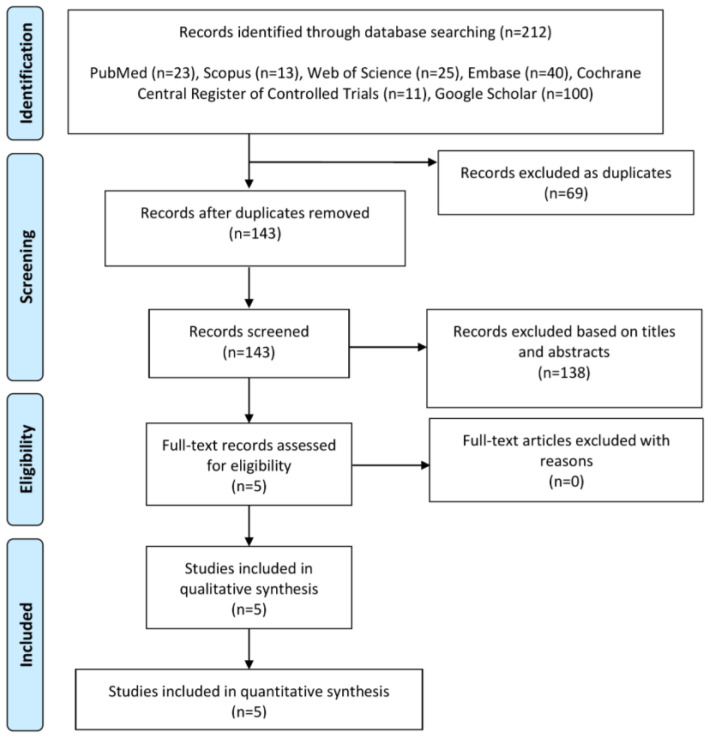
The PRISMA flowchart of literature search.

Figure 1 illustrates the PRISMA flow diagram. Overall, there were four RCTs [3,7,8,9] and one NCT [1] that met the eligibility criteria. These studies comprised 309 patients (SHP block = 156 patients and no SHP block = 153 patients). These studies were published between 2017 and 2020, and conducted in Turkey [1], Pakistan [8], Egypt [7], India [9], and Sweden [3]. The sample size of participants in experimental and control arms ranged from 30 to 35 patients. All SHP blocks were conducted at the end of the abdominal hysterectomy procedure using either bupivacaine (volume range: 20–30 mL, concentration: 0.25%) [1,9] or ropivacaine (volume range: 20–30 mL, concentration range: 0.25–0.75%) [3,7,8]. The type of control was placebo and no intervention in three [3,7,8] and two [1,9] studies, respectively. All studies used perioperative multimodal analgesia irrespective of the administration of SHP block. Supplementary Table S2 depicts a summary of the included studies.

### 3.2. Summary of Risk of Bias of the Included Studies

Supplementary Figure S1 depicts the risk of bias summary of the four RCTs. Overall, three RCTs [3,7,8] showed low risk of bias in all domains. Nonetheless, one RCT (Subramanian 2019) [9] was single-blinded, consequently a judgment of high risk was assigned to the performance bias domain. Additionally, the same RCT (Subramanian 2019) [9] did not offer enough information about outcome assessment, hence the domain of detection bias was scored as unclear risk. Supplementary Table S3 depicts the good quality (i.e., eight stars) assessment of the NCT study (Aytuluk 2020) [1].

### 3.3. Summary of Primary Endpoints

#### 3.3.1. Postsurgical Pain Score

**Figure 2:**
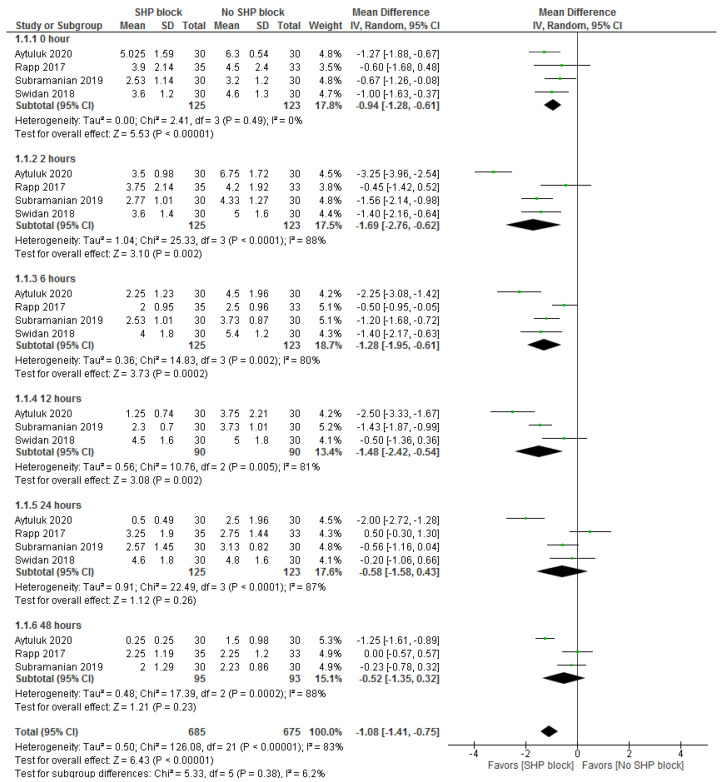
Meta-analysis for postsurgical pain score based on the 10-point visual analogue scale system.

Five studies were meta-analyzed [1,3,7,8,9]. The overall postsurgical pain score was significantly reduced in the SHP block arm contrasted with the control arm (n = 5 studies, MD = −1.08, 95% CI [−1.41, −0.75], *p* < 0.001). The pooled analysis was heterogeneous (I^2^ = 83%, *p* < 0.001). Subgroup analysis based on the postsurgical time point revealed that the effect size was significantly decreased in the SHP block arm contrasted with the control arm at 0 h (n = 4 studies, MD = −0.94, 95% CI [−1.28, −0.61], *p* < 0.001), 2 h (MD = −1.69, 95 CI [−2.76, −0.62], *p* = 0.002), 6 h (n = 4 studies, MD = −1.28, 95% CI [−1.95, −0.61], *p* < 0.001), and 12 h (n = 3 studies, MD = −1.48, 95% CI [−2.42, −0.54], *p* = 0.002) after surgery. Nonetheless, there was no significant variance between both arms pertaining to postsurgical pain at 24 h (n = 4 studies, MD = −0.58, 95% CI [−1.58, 0.43], *p* = 0.26) and 48 h (n = 3 studies, MD = −0.52, 95% CI [−1.35, 0.32], *p* = 0.23) after surgery. While the pooled analysis was homogenous at 0 h (I^2^ = 0%, *p* = 0.49), it was heterogeneous for the other remaining time points (I^2^ > 50%, *p* < 0.1) (Figure 2).

#### 3.3.2. Postsurgical Opioid Consumption

**Figure 3:**
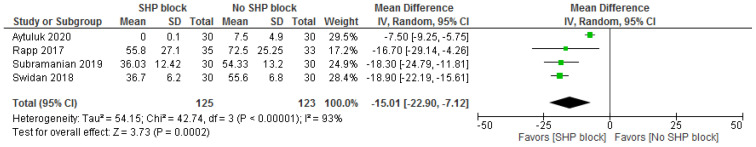
Meta-analysis for postsurgical opioid consumption based on the Morphine Milligram Equivalent (MME) unit.

Four studies were meta-analyzed [1,3,7,9]. The overall postsurgical opioid consumption was significantly decreased in the SHP block arm contrasted with the control arm (n = 4 studies, MD = −18.90 MME, 95% CI, [−22.19, −15.61], *p* < 0.001). The pooled analysis was heterogeneous (I^2^ = 93%, *p* < 0.001) (Figure 3). One RCT (Mahmood 2018) [8] showed that the overall postsurgical opioid consumption of nalbuphine was drastically lowered in the SHP block arm contrasted with the control arm (median (range) values: 3.15 mg [0.67] and 3.80 mg [0.81], respectively; *p* < 0.01).

### 3.4. Summary of Secondary Endpoints

#### 3.4.1. Postsurgical NSAID Consumption

**Figure 4:**
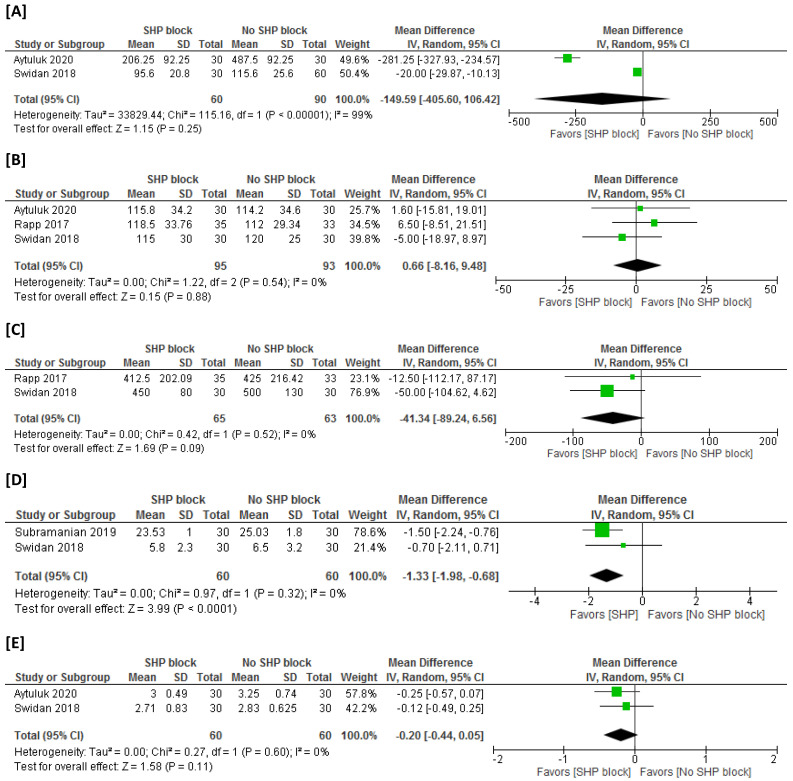

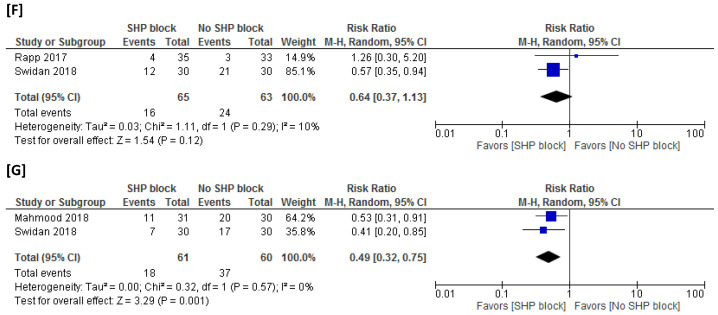
Meta-analysis for secondary endpoints: (**A**) postsurgical NSAID consumption (mg), (**B**) operation time (min), (**C**) estimated intraoperative blood loss (mL), (**D**) time to first mobilization (h), (**E**) length of hospital stay (d), (**F**) rate of postoperative nausea (%), and (**G**) rate of postoperative vomiting (%).

Two studies were meta-analyzed [1,7]. One study (Aytuluk 2020) [1] depicted a significant reduction in postsurgical NSAID consumption with the use of SHP block. The second study (Swidan 2018) [7] also demonstrated similar reduction in postsurgical NSAID consumption with the use of SHP block; however, the extent of reduction was not as dramatic as the first study. Nevertheless, overall, the pooled meta-analysis findings discovered no significant difference in postsurgical NSAID consumption between both arms (n = 2 studies, MD = −149.59 mg, 95% CI [−405.60, 106.42], *p* = 0.25). The pooled analysis was heterogeneous (I^2^ = 99%, *p* < 0.001) (Figure 4A).

#### 3.4.2. Rescue Analgesic Time

One study (Aytuluk 2020) [1] showed the mean ± SD rescue analgesic time was significantly longer for the SHP block group compared with the control group: 627 ± 352.9 min vs. 203.8 ± 173.1 min, respectively; *p* < 0.001.

#### 3.4.3. Operation Time

Three studies were meta-analyzed [1,3,7]. There was no significant difference in mean operation time between both groups (n = 3 studies, MD = 0.66 min, 95% CI [−8.16, 9.48], *p* = 0.88). The pooled analysis was homogeneous (I^2^ = 0%, *p* = 0.54) (Figure 4B).

#### 3.4.4. Estimated Intraoperative Blood Loss

Two studies were meta-analyzed [3,7]. There was no significant difference in estimated intraoperative blood loss between both groups (n = 2 studies, MD = −41.34 mL, 95% CI [−89.24, 6.56], *p* = 0.09). The pooled analysis was homogeneous (I^2^ = 0%, *p* = 0.52) (Figure 4C).

#### 3.4.5. Time to First Mobilization

Two studies were meta-analyzed [7,9]. The mean time to first mobilization after surgery was significantly shorter in favor of the SHP block group compared with the control group (n = 2 studies, MD = −1.33 h, 95% CI [−1.98, −0.68], *p* < 0.001). The pooled analysis was homogeneous (I^2^ = 0%, *p* = 0.32) (Figure 4D). However, one study (Rapp 2017) [3] showed that rate of patients who mobilized after surgery was comparable and did not significantly differ between both groups (90% in both groups, *p* = 1.0).

#### 3.4.6. Time to First Bowel Movement and Urinary Passage

Swidan et al. [7] demonstrated that the mean ± SD time to first flatus was significantly shorter in the SHP block arm compared with the control arm: 38 ± 6 h vs. 42 ± 5 h, respectively; *p* = 0.0068. However, on postoperative day 1, Rapp and colleagues [3] displayed that the frequency of patients who had bowel movement was not significantly different between both arms (35% and 37% in the SHP block and control groups, respectively; *p* = 0.72). After elimination of urinary catheter at 24 h postoperation, Subramanian et al. [9] showed that the mean ± SD time to first urinary passage was not significantly different between SHP block and control arms: 3.02 ± 1 h vs. 3.05 ± 0.67 h, respectively; *p* = 0.738.

#### 3.4.7. Length of Hospital Stay

Two studies were meta-analyzed [1,7]. The results depicted no significant difference between arms (n = 2 studies, MD = −0.20 d, 95% CI [−0.44, 0.05], *p* = 0.11). The pooled analysis was homogeneous (I^2^ = 0%, *p* = 0.54) (Figure 4E).

#### 3.4.8. Adverse Events

Two studies reported outcomes for postoperative nausea and vomiting independently [3,7]. While the summary results displayed that the frequency of postsurgical nausea was not significantly different between both arms (n = 2 studies, RR = 0.64, 95% CI [0.37, 1.13], *p* = 0.12) (Figure 4F), the frequency of postsurgical vomiting was significantly reduced in favor of the SHP block group contrasted with the control arm (n = 2 studies, RR = 0.49, 95% CI [0.32, 0.75], *p* = 0.001) (Figure 4G). The pooled analyses were homogenous (I^2^ = 10%, *p* = 0.29 and I^2^ = 0%, *p* = 0.57, respectively). Aytuluk et al. [1] reported that frequency of combined postsurgical nausea and vomiting (PONV) was not significantly different between both arms (23.3% vs. 26.7%, *p* = 0.766). Subramanian et al. [9] documented that were was no significant adverse events observed intraoperatively and postoperatively in both groups. Rapp et al. [3] reported two intraoperative incidents comprising minor intraoperative injury to urinary bladder (n = 1) and blood loss more than 1000 mL (n = 1) in the SHP block group who received saline, both of which were not directly related to SHP intervention. None of the reviewed studies recorded sympathetic block-related adverse events, such as bradycardia and hypotensive incidents.

### 3.5. Leave-One-Out Sensitivity Analysis for the Primary Endpoints

Leave-one-out sensitivity analysis confirmed the robustness of all endpoints, except the postsurgical pain score at 12 h. The omission of Subramanian’s 2019 study [9] for postsurgical pain score at 12 h impacted the overall effect size resulting in an insignificant difference between both groups (n = 2 studies, MD = −1.50, 95% CI [−3.46, 0.46], *p* = 0.133) (Supplementary Figures S2A–F and S3).

### 3.6. Publication Bias Analysis for the Primary Endpoints

Supplementary Figures S4A–F and S5 depict the funnel plot-based publication bias analysis. All endpoints showed no publication bias based on Egger’s regression test, except postsurgical pain score at 48 h (*p* < 0.001).

## 4. Discussion

### 4.1. Summary of Results

This investigation was performed to delineate the analgesic benefits and morbidity of SHP block (intervention) compared with no SHP block (control) during abdominal hysterectomy. Four RCTs and one NCT with 210 patients were included in this investigation. The studies showed low risk of bias and exhibited good quality. The findings revealed that the SHP block appeared safe and decreased postsurgical pain, opioid intake, and immobilization time. However, SHP block did not appear to associate with clinical benefits pertaining to reductions in duration of the surgery, amount of intraoperative bleeding, and length of hospitalization in comparison with the control group. Sensitivity analysis showed robustness of the primary endpoints without publication bias.

### 4.2. Explanation of Results and Clinical Implications

Sufficient handling of postsurgical pain after abdominal hysterectomy is an imperative endpoint, as unsatisfactory management of acute postsurgical pain is coupled with a large number of unfavorable sequelae. Examples of such sequelae encompass postponed mobilization, delayed functional recovery, increased healthcare expenses, and poor quality of life [2]. A further noteworthy sequela includes the chronic and excessive intake of NSAIDs and opioids [1,2]. NSAIDS are associated with various gastrointestinal, cardiovascular, and renal side effects [19]. On the other hand, chronic intake of opioids is associated with serious complications, most notably respiratory depression and long-term opioid addiction [20]. In fact, opioid-free multimodal analgesic schemes to lessen postsurgical pain and hasten functional recovery are increasingly endorsed [2]. All studies in this meta-analysis used perioperative multimodal analgesia irrespective of the administration of SHP block. Postsurgical pain after abdominal hysterectomy can be evaluated subjectively via patient-reported pain grades and objectively via postoperative opioid intake. In comparison with the absence of SHP block, the present investigation documented that SHP block was linked to improved pain-reliving outcomes, reflected by smaller VAS pain grades and opioid intake. The magnitude of the subjective reduced pain score was statistically significant and clinically meaningful, as reflected by reductions of ≥1 point out of the 10-point VAS [21]. These findings highlight the clinical implication of SHP block as a valuable opioid-free multimodal analgesic intervention during abdominal hysterectomy.

Several lines of investigations documented the analgesic benefits of SHP to patients with enduring pelvic pain, inclusive of malignant- (e.g., cancer) and benign (e.g., endometriosis)-related etiologies [6,22]. Within the fields of obstetrics and gynecology, SHP block has been displayed to adequately control postsurgical pain among women undergoing cesarean section [23,24], uterine artery embolization [25], and laparoscopic hysterectomy [26,27,28]. This present investigation further inflates the utilization landscape of the SHP block to incorporate a rationale for postsurgical pain relief after abdominal hysterectomy.

SHP block is traditionally accomplished with the guidance of an imaging modality, for example, ultrasonography. Nonetheless, during abdominal hysterectomy, the intraabdominal as well as pelvic structures are well-visualized intraoperatively. Therefore, the SHP can be quickly, easily, and directly accessed, even without the necessity for an imaging-based guidance [1,3]. Aytuluk et al. [1] reported that fluoroscopy was used during the initial experience of SHP block to guide the needle position and contrast spread. However, the use of fluoroscopy was later omitted and deemed unnecessary. All in all, the application of SHP block during abdominal hysterectomy is very reasonable, fast to execute without prolonging duration of surgery, and does not mandatorily require use of imaging guidance.

From an anatomical point of view, the SHP is positioned next to important structures, for example, small/large colon, urinary bladder, vertebral column, and somatic nerves. Therefore, intraoperative iatrogenic injuries are possible aftermaths. Additionally, hypotension and bradycardia are possible consequences of the sympathetic block of the SHP [29]. Collectively, the current investigation highlights the intraoperative and postoperative safety of the SHP block during abdominal hysterectomy.

There are several clinical implications that should be highlighted from the present investigation. Ropivacaine and bupivacaine are common agents used in SHP block. While both agents have relatively equal potency, the safety profile of ropivacaine is relatively better than bupivacaine [30,31]. All meta-analyzed studies [1,3,7,8,9] have performed the SHP block toward the end of the operation. This timing point is critical as it can favorably guarantee adequacy of analgesia during the early postsurgical period. Injection of higher volumes of local anesthetic (i.e., ≥20 mL) during SHP block is preferred and linked to better analgesic effects compared with lower volumes [32,33].

### 4.3. Comparsion with Previous Meta-Analysis Reports

Alomar and colleagues [34] examined the role of SHP block versus none during minimally invasive hysterectomy. The authors included three studies and concluded the morbidity-free and satisfactory postoperative pain-reliving and opioid-free effects of SHP block. Shama and partners [35] evaluated the role of SHP block in yielding satisfactory pain control during hysterectomy. The authors concluded that SHP effectively reduced postsurgical pain, opioid intake, and frequency of vomiting/nausea postoperatively. However, the above-mentioned meta-analysis included only four mixed studies (n = 3 abdominal hysterectomy and n = 1 laparoscopic hysterectomy) and reported very few endpoints compared with our meta-analysis. Additionally, the authors did not perform examination for robustness or publication bias.

### 4.4. Strengths and Weaknesses

Our investigation harbors numerous strengths. Most notably, we reported the first ever review report to probe the effectiveness of SHP block in controlling postsurgical pain after abdominal hysterectomy. In our analysis, we considered both randomized and nonrandomized investigations to augment the power of the assembled deductions, which is an endorsed recommendation [36,37]. Moreover, we conducted a PRISMA-complaint investigation to ensure high-quality reporting of findings. Moreover, we analyzed quantitatively and qualitatively many primary and secondary endpoints. For the primary endpoints, we further examined their stability based on sensitivity analysis and explored their risk for publication predisposition.

Nevertheless, the present research equally harbors some weaknesses. The foremost weakness lies in the small number of studies and their sample sizes. Another weakness embraces the existence of heterogeneity in study designs (i.e., RCTs vs. nonrandomized comparative trials) and surgical procedures (e.g., volume of injected amide local anesthetic). Accordingly, these factors could have in some way influenced the pooled summary effect sizes. Although one study (Subramanian 2019) [9] was not double-blinded, the primary endpoints were not considerably influenced by this blinding deficiency. Additional limitations include the meta-analytical pooling of secondary endpoints from two studies only. Lastly, in consideration of the small number of studies per outcome (i.e., <10 studies), the results of publication bias (including Egger linear regression test) should be interpreted with caution [17].

### 4.5. Future Directions

Important future directions comprise the need for additional large-sized RCTs to authenticate the findings of the present research. The root of postsurgical pain after abdominal hysterectomy may be credited to visceral and somatic pain origins [3]. Therefore, a considerable future direction includes examining the synergetic analgesic efficacy of SHP block and abdominal wall plane block to lessen the visceral and somatic pain origins, respectively. In addition to SHP block, enrollment of patients in Enhanced Recovery After Surgery (ERAS) protocols is expected to further heighten the analgesic and postoperative outcomes [38,39]. Future exploration may investigate the best amide anesthetic (i.e., bupivacaine compared with ropivacaine) for SHP block during abdominal hysterectomy, in addition to examining the related pharmacodynamics. Moreover, it is worthwhile to recognize the clusters of patients who are projected to attain greater benefits from the application of SHP block during abdominal hysterectomy. Finally, it is important to inspect the analgesic effects of SHP block among patients undergoing relatively less traumatic hysterectomy approaches, such as robotic hysterectomy [40].

## 5. Conclusions

During abdominal hysterectomy, this investigation revealed that intraoperative SHP block seemed safe and decreased postsurgical pain, opioid intake, and immobilization time. However, SHP block did not appear to associate with clinical benefits pertaining to reductions in duration of surgery, amount of intraoperative bleeding, and length of hospitalization in comparison with the control group.

## Data Availability

All data are available in the manuscript and its supplementary files.

## Supplementary Materials

The following supporting information can be downloaded at: https://www.mdpi.com/article/10.3390/medicina59050893/s1, Supplementary Table S1. The exact query search strategy used in all information sources; Supplementary Table S2. The baseline characteristics of the included studies; Supplementary Table S3. Quality assessment according to the Newcastle–Ottawa Scale for cohort studies; Supplementary Figure S1. Risk of bias assessment of the randomized controlled trials; Supplementary Figure S2. Leave-one-out sensitivity analysis for postsurgical pain score based on the 10-point visual analogue scale at 0 h [A], 2 h [B], 6 h [C], 12 h [D], 24 h [E], and 48 h [F]; Supplementary Figure S3. Leave-one-out sensitivity analysis for postsurgical opioid consumption based on the Morphine Milligram Equivalent (MME) unit; Supplementary Figure S4. Funnel plot-based publication bias analysis for postsurgical pain score based on the 10-point visual analogue scale at 0 h [A], 2 h [B], 6 h [C], 12 h [D], 24 h [E], and 48 h [F]; Supplementary Figure S5. Funnel plot-based publication bias analysis for postsurgical opioid consumption based on the Morphine Milligram Equivalent (MME) unit.
