# Supplementary material for: Analgesic Efficacy of Intraoperative Superior Hypogastric Plexus (SHP) Block during Abdominal Hysterectomy: A Systematic Review and Meta-Analysis of Controlled Trials
Source: Medicina (Kaunas). 2023 May 6;59(5):893. doi: 10.3390/medicina59050893 (PMC10220697; doi:10.3390/medicina59050893)

Supplementary Table S1. The exact query search strategy used in all information sources.

**PubMed**<br>All Fields: (superior hypogastric plexus OR SHP OR presacral plexus OR presacral nerve) AND (block OR neurolysis OR neurectomy) AND (hysterectomy)
**Scopus**<br>TITLE-ABS-KEY (({Superior hypogastric plexus} OR SHP OR {presacral plexus} OR {presacral nerve}) AND (block OR neurolysis OR neurectomy) AND (hysterectomy))
**Web of Science**<br>All Fields: (superior hypogastric plexus OR SHP OR presacral plexus OR presacral nerve) AND (block OR neurolysis OR neurectomy) AND (hysterectomy)
**Embase**<br>Quick search: ('superior hypogastric plexus'/exp OR 'superior hypogastric plexus' OR (superior AND hypogastric AND ('plexus'/exp OR plexus)) OR shp OR 'presacral plexus' OR (presacral AND ('plexus'/exp OR plexus)) OR 'presacral nerve' OR (presacral AND ('nerve'/exp OR nerve))) AND (block OR 'neurolysis'/exp OR neurolysis OR 'neurectomy'/exp OR neurectomy) AND ('hysterectomy'/exp OR hysterectomy)
**Cochrane Central Register of Controlled Trials (CENTRAL)**<br>Title Abstract Keyword: (superior hypogastric plexus OR SHP OR presacral plexus OR presacral nerve) AND (block OR neurolysis OR neurectomy) AND (hysterectomy)
**Google Scholar**<br>All Fields: (superior hypogastric plexus OR SHP OR presacral plexus OR presacral nerve) AND (block OR neurolysis OR neurectomy) AND (hysterectomy)

Supplementary Table S2. The baseline characteristics of the included studies.

Study ID [Author, year]	Country	Study design	Groups	Details of interventions	n	Age in years	BMI in kg/m²
Aytuluk 2020	Turkey	NCT	Experimental	SHP block: Performed at the end of the surgery with 30 mL 0.25% bupivacaine	30	52.4 ± 6.5	29.4 ± 4.7
			Control	No-SHP block: No intervention	30	51.6 ± 7.5	29.37 ± 3.93
Mahmood 2018	Pakistan	RCT	Experimental	SHP block: Performed at the end of the surgery with 20 mL 0.25% ropivacaine	31	NR*	23 (14)
			Control	No-SHP block: Performed at the end of the surgery with 20 mL saline	30	NR*	23 (17)
Rapp 2017	Sweden	RCT	Experimental	SHP block: Performed at the end of the surgery with 20 mL 0.75% ropivacaine	35	46.0 (35–63)	NR**
			Control	No-SHP block: Performed at the end of the surgery with 20 mL saline	33	45.5 (34–69)	NR**
Subramanian 2019	India	RCT	Experimental	SHP block: Performed at the end of the surgery with 20-25 mL 0.25% bupivacaine	30	40.47 ± 9.29	40.53 ± 7.25
			Control	No-SHP block: No intervention	30	40.53 ± 7.25	23.39 ± 3.18
Swidan 2017	Egypt	RCT	Experimental	SHP block: Performed at the end of the surgery with 20 mL 0.5% ropivacaine	30	48.3 ± 5.7	30.2 ± 14.2
			Control	No-SHP block: Performed at the end of the surgery with 20 mL saline	30	49.2 ±14.9	31.2 ± 5.1

Abbreviations—BMI: body mass index; NR: not reported; NCS: nonrandomized comparative trial; RCT: randomized controlled trial; SHP: superior hypogastric plexus
Age and BMI were reported as mean ± standard deviation, median (range), or median (minimum–maximum).
* For Mahmood 2018 study, the proportions of experimental patients with age range 35-40, 41-50, 51-55, and 56-60 years were 12.9 (n=4), 61.3% (n=16.1), 16.1% (n=5) and 9.7% (n=3). The proportions of experimental patients with age range 35-40, 41-50, 51-55, and 56-60 year were 13.3% (n=4), 50% (n=15), 13.3% (n=4), and 23.3% (n=7).
** For Rapp 2017 study, the proportions of experimental patients with BMI ≤25, BMI >25 and <30, and BMI ≥30 kg/m² were 34.3% (n=12), 34.3% (n=12), and 31.4% (n=11). The proportions of control patients with BMI ≤25, BMI >25 and <30, and BMI ≥30 were 48.5% (n=16), 27.3% (n=9), and 24.2% (n=8).

Supplementary Table S3. Quality assessment according to the Newcastle-Ottawa Scale for cohort studies.

Items	Selection				Comparability		Outcome			Overall score
	Representativeness of the exposed cohort	Selection of the non-exposed cohort	Ascertainment of exposure	Demonstration that outcome of interest was not present at start of study	The study controls for demographics	The study controls for randomization	Assessment of outcome	Was follow-up long enough for outcomes to occur	Adequacy of follow-up of cohorts	
Aytuluk 2020	*	*	*	*	*		*	*	*	8/9

Supplementary Figure S1. Risk of bias assessment of the randomized controlled trials.

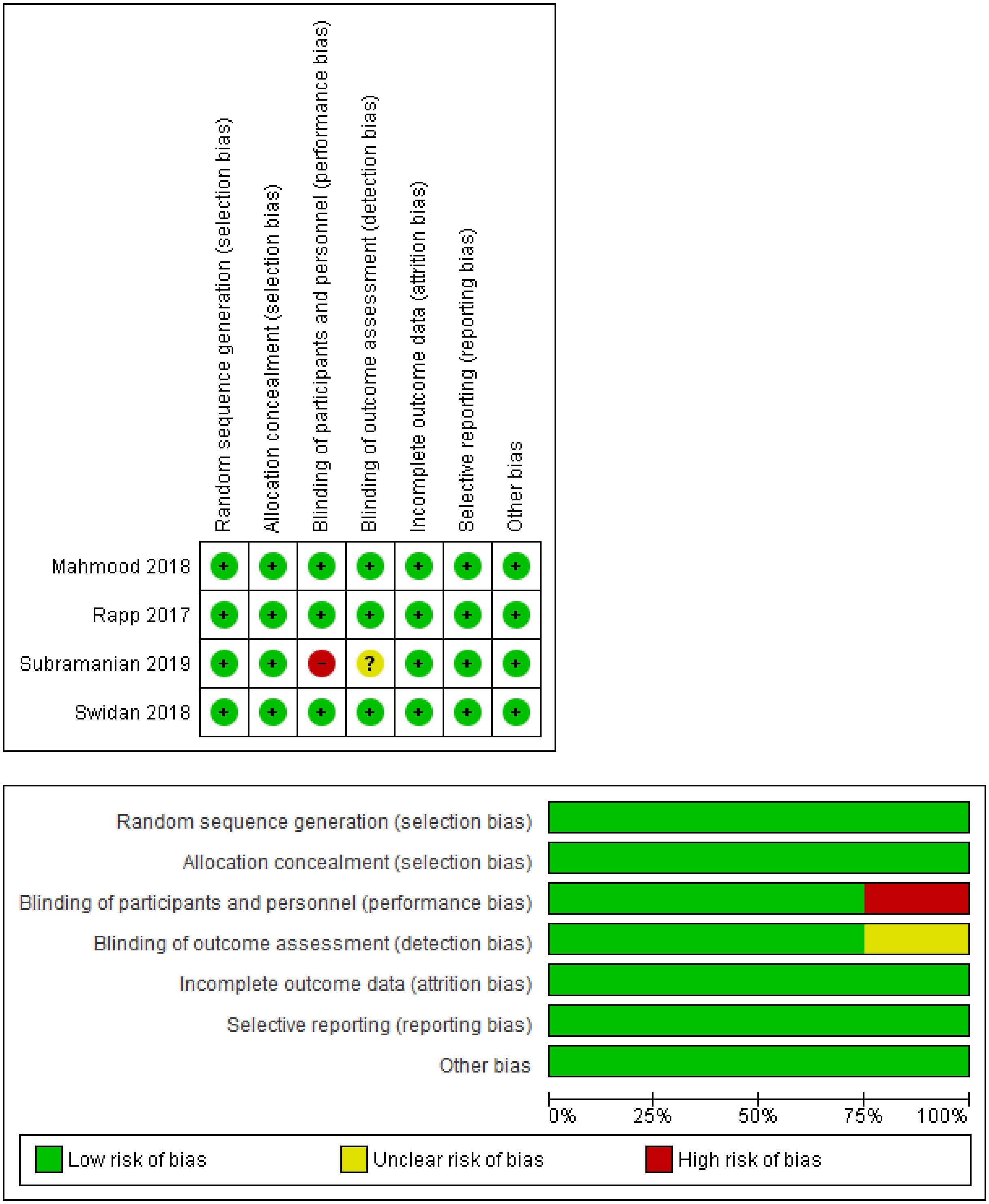

Supplementary Figure S2. Leave-one-out sensitivity analysis for postsurgical pain score based on the 10-point visual analogue scale at 0 hour **[A]**, 2 hours **[B]**, 6 hours **[C]**, 12 hours **[D]**, 24 hours **[E]**, and 48 hours **[F]**.

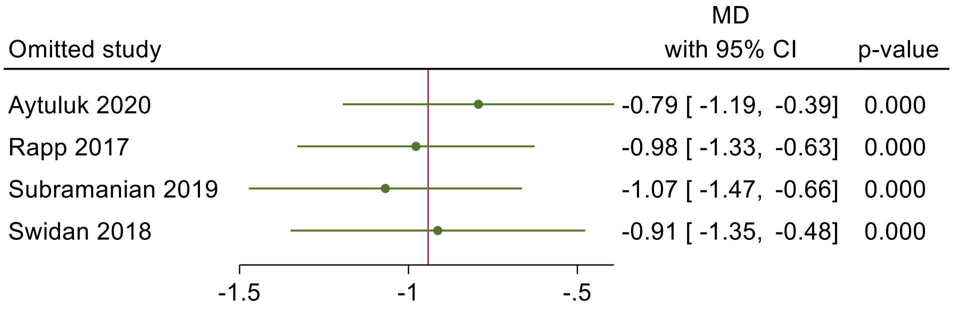

[B]

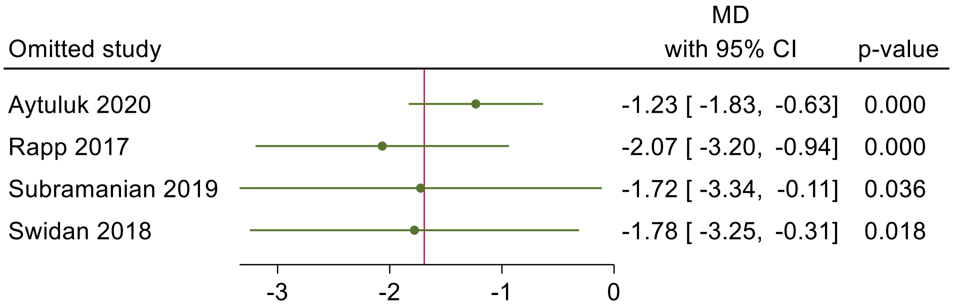

[C]

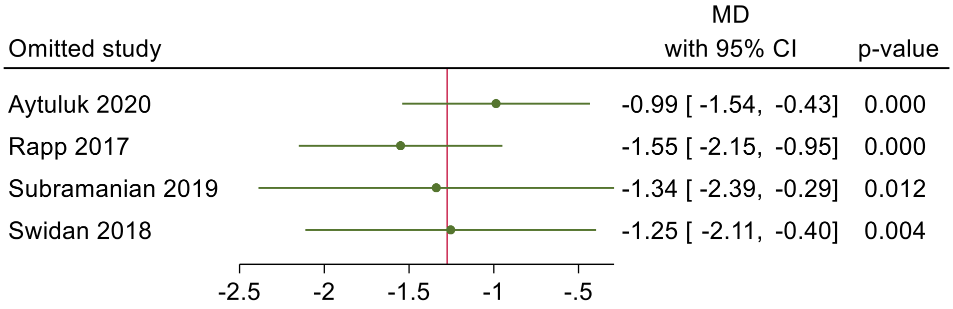

[D]

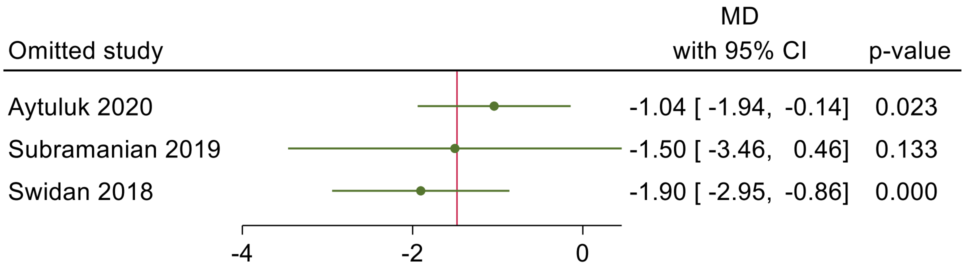

[E]

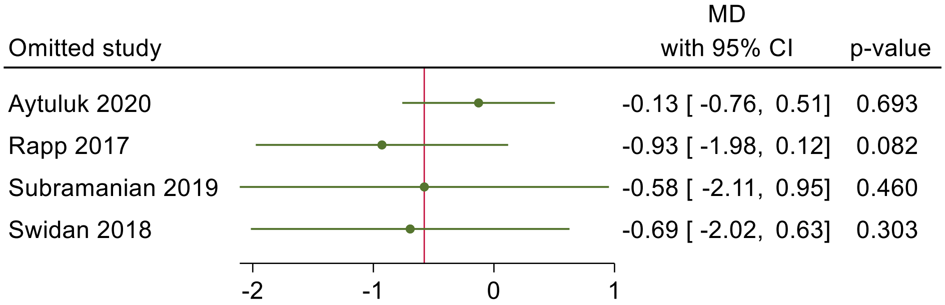
MD
with 95% CI
Omitted study
p-value
Aytuluk 2020
-0.13 [-0.76, 0.51]
0.693
Rapp 2017
-0.93 [-1.98, 0.12]
0.082
Subramanian 2019
-0.58 [-2.11, 0.95]
0.460
Swidan 2018
-0.69 [-2.02, 0.63]
0.303
-2
-1
0
1

[F]

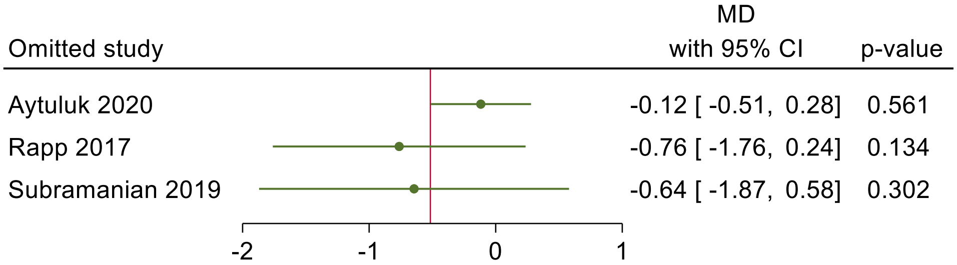
MD
with 95% CI
Omitted study
p-value
Aytuluk 2020
-0.12 [-0.51, 0.28]
0.561
Rapp 2017
-0.76 [-1.76, 0.24]
0.134
Subramanian 2019
-0.64 [-1.87, 0.58]
0.302
-2
-1
0
1

Supplementary Figure S3. Leave-one-out sensitivity analysis for postsurgical opioid consumption based on the Morphine Milligram Equivalent (MME) unit.

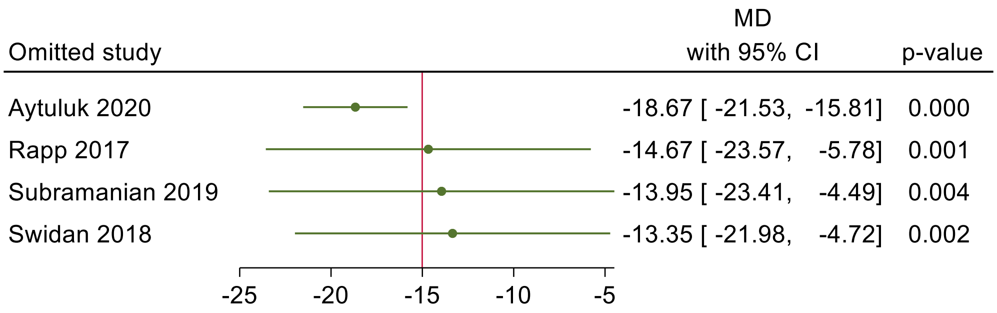

Supplementary Figure S4. Funnel plot-based publication bias analysis for postsurgical pain score based on the 10-point visual analogue scale at 0 hour **[A]**, 2 hours **[B]**, 6 hours **[C]**, 12 hours **[D]**, 24 hours **[E]**, and 48 hours **[F]**.

[A]

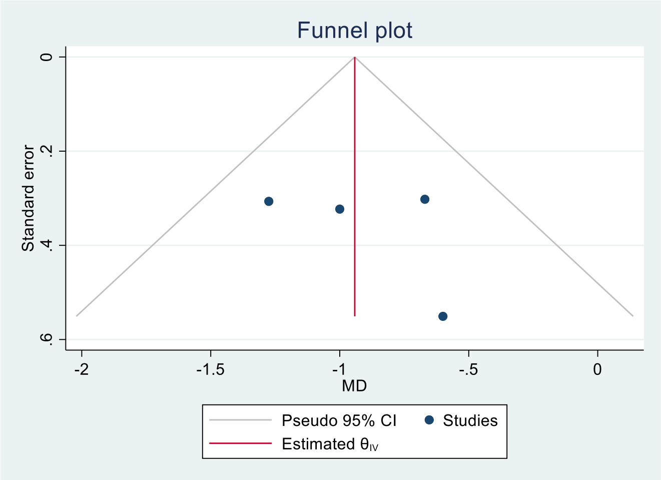

[B]

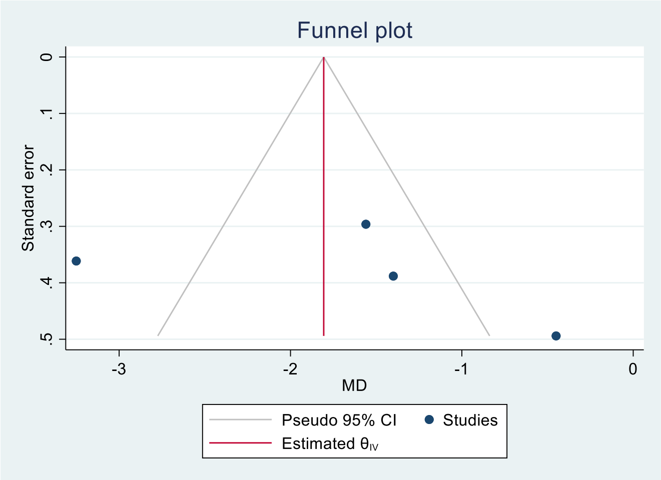

[C]

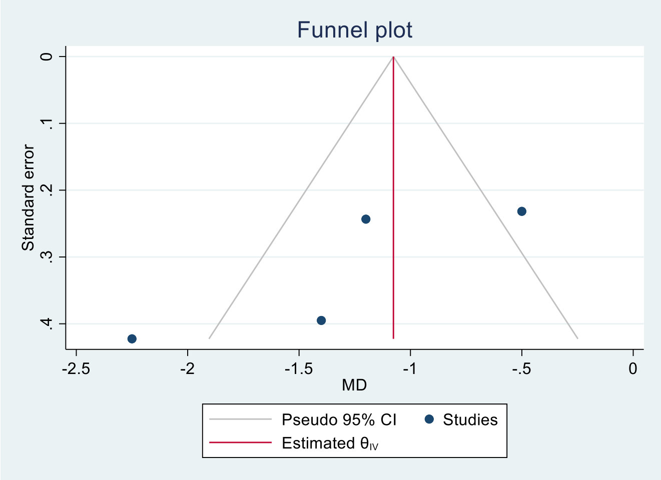

[D]

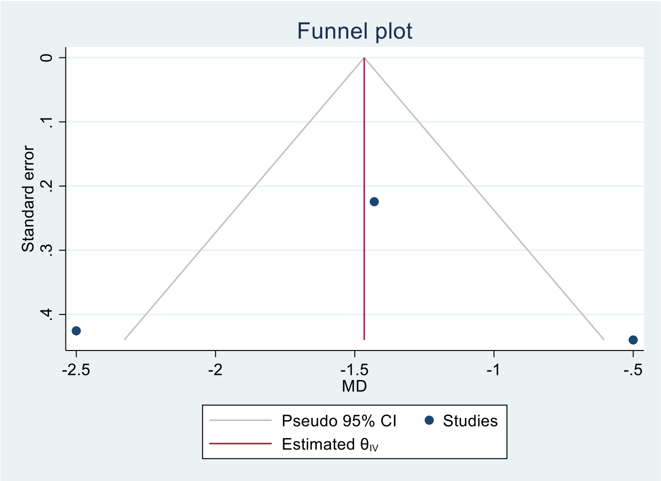
Funnel plot
Standard error
0
.1
.2
.3
.4
-2.5
-2
-1.5
-1
-.5
MD
Pseudo 95% CI
Studies
Estimated θIV

[E]

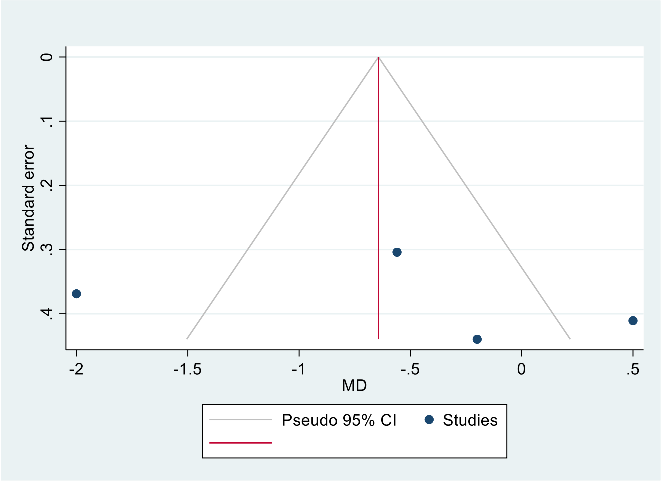
Standard error
0
.1
.2
.3
.4
-2
-1.5
-1
-.5
0
.5
MD
Pseudo 95% CI
Studies

[F]

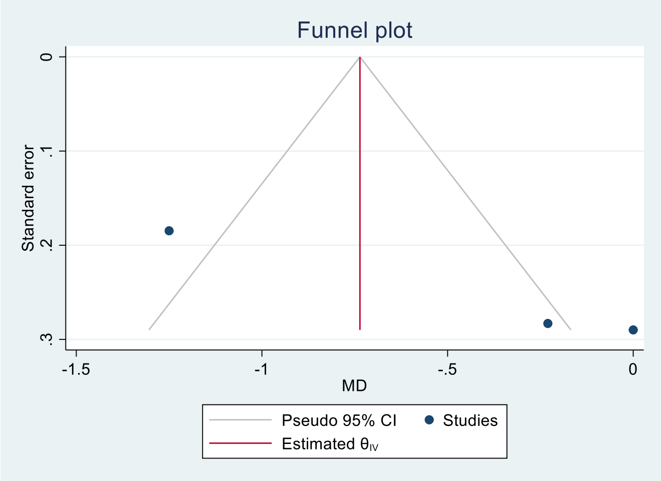
Funnel plot
Standard error
0
.1
.2
.3
-1.5
-1
-.5
0
MD
Pseudo 95% CI
Studies
Estimated θIV

Supplementary Figure S5. Funnel plot-based publication bias analysis for postsurgical opioid consumption based on the Morphine Milligram Equivalent (MME) unit.

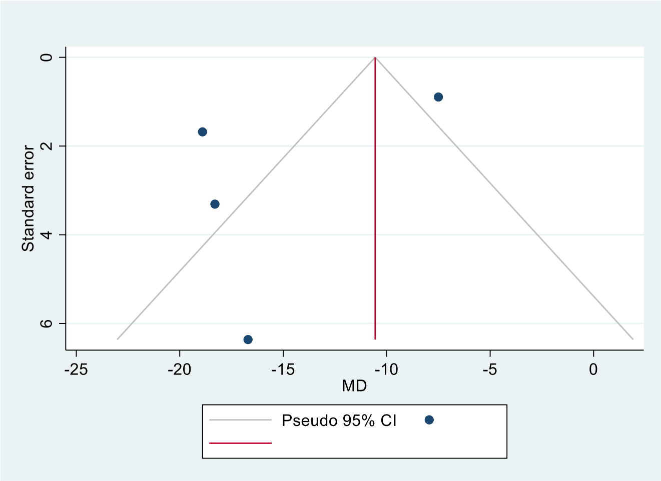

Supplement: Supplementary file 1 [file medicina-59-00893-s001.zip › medicina-2308581-supplementary.pdf]
